# Effects of Iron Salts on Demineralization and Discoloration of Primary Incisor Enamel Subjected to Artificial Cariogenic Challenge versus Saline Immersion

**DOI:** 10.3390/healthcare11040569

**Published:** 2023-02-14

**Authors:** Bahareh Nazemisalman, Mehran Mohseni, Shayan Darvish, Mahya Farsadeghi, Ionut Luchian

**Affiliations:** 1 Department of Pediatric Dentistry, School of Dentistry, Zanjan University of Medical Sciences, Zanjan 4513956184, Iran; 2 Food and Drug Control Department, Zanjan University of Medical Sciences, Zanjan 4513956184, Iran; 3 Faculty of Dentistry, University of British Columbia, Vancouver, BC V6T 1Z3, Canada; 4 School of Dentistry, Zanjan University of Medical Sciences, Zanjan 4513956184, Iran; 5 Department of Periodontology, Faculty of Dental Medicine, “Grigore T. Popa” University of Medicine and Pharmacy, 700115 Iasi, Romania

**Keywords:** iron compounds, deciduous tooth, anemia, iron-deficiency

## Abstract

Aim: This study aimed to assess the effects of iron salts on the demineralization and discoloration of primary incisor enamel subjected to artificial cariogenic challenge (ACC) versus saline immersion. Methodology: In this in vitro experimental study, 90 primary incisors were evaluated in 10 groups (*n* = 9). Five groups were subjected to ACC, and the other five were immersed in saline. Ferrous sulfate, ferrous fumarate, ferrous ammonium citrate, and ferrous gluconate were added to both saline and cariogenic solutions. The solutions were refreshed every 48 h. After 14 days, the teeth were removed from the media and their demineralization was inspected via scanning electron microscopy (SEM). Energy-dispersive X-ray spectroscopy (EDX) was also performed. The color of the specimens was measured at baseline and after the intervention using the Vita Shade Guide. Results: Data were analyzed by the Kruskal–Wallis test, one-way ANOVA, and Tukey’s test. The color change of specimens subjected to ACC was greater than the color change of those in saline (*p* = 0.083). The teeth subjected to ACC showed greater iron uptake than did those in saline (*p* = 0.023). SEM assessment revealed a regular pattern of enamel prisms, with some broken prisms and superficial cracks in the teeth immersed in saline. The teeth subjected to ACC showed numerous fractures and cracks, which were greater in the ferrous sulfate group. Conclusions: Immersion in ACC increased the structural porosities and led to greater iron uptake and, consequently, higher discoloration. The maximum structural changes and subsequent staining were noted in the ferrous sulfate group, followed by ferrous ammonium citrate, ferrous fumarate, and ferrous gluconate.

## 1. Introduction

Physical and psychological growth and development is a dynamic process that greatly depends on proper nutrition [1]. A low-iron diet can cause serious behavioral, functional, and cognitive problems in children, and evidence shows that iron-deficient children often show inferior performance in terms of intelligence and motor functions compared to their normal peers [2]. Iron supplements are commonly prescribed to prevent iron deficiency anemia; however, tooth discoloration is a major side effect [3]. Parents are often concerned about black discoloration of the teeth due to iron drops and commonly seek dental treatment. Many parents mistake iron staining for dental caries or believe that iron supplements have resulted in the development of dental caries and decide to discontinue it. This will lead to early iron deficiency anemia in children, and the adverse consequences may remain for years [4]. Insoluble iron compounds react with the gingival crevicular fluid and subgingival bacterial metabolites and produce sulfur-containing compounds, such as hydrogen sulfide, which is responsible for tooth discoloration. Considering the high levels of this metabolite in children with poor oral hygiene or enamel defects, such children are more susceptible to greater tooth discoloration [5]. In addition, sweeteners added to the available iron drops in the Iranian market often contain ferrous sulfate due to their unfavorable taste. These sweeteners may also cause dental caries [6].

The possible effects of iron regarding caries development, enamel decalcification, saliva viscosity, tooth staining, and oral microbial flora have been extensively investigated, with controversial results [7]. Shojaipour et al. indicated black discoloration of teeth due to iron drop consumption [8]. Similar results were reported by Christofides et al. [9] and Pushoanjali et al. [10]. Ellingsen et al. [11] and Reid et al. [12] discussed that ferrous sulfate is an extrinsic factor causing tooth discoloration, whereas Addy et al. [13] reported that use of metal salts did not cause tooth discoloration. According to them, the exact mechanism of metal binding salts to tooth surfaces is not clearly understood, and it seems that some superficial changes occur as a result of interactions with the acquired pellicle.

Considering the significance of using iron drops and the gap of information regarding the structural changes of primary enamel following exposure to iron salts, this study aimed to assess the effect of four commonly consumed iron salts on the demineralization and discoloration of primary incisor enamel subjected to artificial cariogenic challenge (ACC) and saline immersion.

## 2. Materials and Methods

### 2.1. Sample Collection and Preparation

This in vitro experimental study was conducted on sound primary central incisors extracted within the past month due to either severe mobility or over-retention.

The sample size was calculated to be 9 in each group, according to a pilot study considering α = 0.10, β = 0.25, and minimum effect size between each two groups to be 1.81. Thus, a total of 90 teeth were enrolled. Teeth with carious lesions, fractures, and enamel structural defects (such as hypoplasia) were excluded. The teeth were stored in saline after extraction, and the saline was refreshed every 48 h.

*S. mutans* (ATCC35668 and PTCC1683) was purchased in lyophilized form from the Iranian Microbial Culture Collection. The frozen vial was rinsed under lukewarm water to defrost. The bacteria were then transferred to blood agar (Liofilchem, Roseto degli Abruzzi, Italy) and incubated in the presence of CO_2_ for 18–24 h. Next, the bacteria were transferred to brain heart infusion broth (Merck, Darmstadt, Germany) under a hood.

For preparation of the solution containing 25 mg iron in 3 cc saline, 228.19 mg of ferrous fumarate, 353.21 mg of ferrous ammonium citrate, 373.2 mg of ferrous sulfate, and 647.6 mg of ferrous gluconate (based on the molecular weight of iron salts and atomic number of iron) were required for each tube (Chimi^®^, Alvand, Iran).

The collected teeth were cleaned with pumice paste and a low-speed handpiece. Next, the root and crown were separated at the cementoenamel junction, and the pulp chamber was sealed with composite resin.

To prepare the artificial cariogenic solution for ACC, 3.7 g of brain heart infusion broth, 0.5 g of extracted yeast (Merck, Germany), 2 g of sucrose (Sigma-Aldrich, Burlington, MA, USA), and 1 g of glucose (Sigma) were dissolved in 100 mL of distilled water; 100 µL of freshly cultured standard-stain (ATCC35668) *S. mutans* (18–24 h) was added to the cariogenic medium, while the pH remained at 4. One test tube was allocated to each specimen.

### 2.2. Grouping

The total of 90 teeth were randomized into 10 groups (*n* = 9). The teeth were subjected to ACC in five groups and immersed in saline in the remaining five groups. Each group subjected to ACC or saline was also exposed to one iron salt.

As explained earlier, in order to achieve 25 mg of iron in each test tube (standard amount of iron in commercial iron drops), 373.2 mg of ferrous sulfate, 228.1 mg of ferrous fumarate, 353.21 mg of ferrous ammonium citrate, and 647.6 mg of ferrous gluconate were required. After weighing using a laboratory precision balance type EW-N/EG-N (Kern & Sohns, Hamburg, Germany), the iron salts were added to the test tubes such that in the first five groups, 200 mL of saline was added to each test tube, and 100 µL of the fresh culture was added to the second five groups. The test tubes were then placed in a Bain–Marie shaker (Biotech, Nordost, Germany) at 37 °C for 10 min. Next, they were incubated at 37 °C for 48 h. To prevent contamination, the tubes were capped. The media were refreshed every 48 h. After 2 weeks, they were removed from the medium and their color was measured and coded using the Vita Shade Guide (Vita Zahnfabrik, Bad Säckingen, Germany), which has 16 color codes from A1 to D4.

The color of the teeth was measured before and after the intervention in a blind manner such that the examiner was not aware of the group allocation of specimens. The teeth were coded as follows:

Score 0: No staining;

Score 1: Slight staining;

Score 2: Heavy staining (out of the range of the Vita Shade Guide).

### 2.3. Structural Assessment

Two teeth were selected randomly from each group (a total of 20) for the scanning electron microscopic (SEM) assessment. The teeth were dried with warm air, gold sputter- coated, and inspected under a scanning electron microscope. In addition, to assess the amount of iron uptake, energy dispersive X-ray spectroscopy (EDX) was performed, which allows identification of constitutional elements semi-quantitatively.

### 2.4. Statistical Analysis

Data were analyzed using SPSS version 22 (IBM Inc., Armonk, NY, USA). Normal distribution of data was evaluated by the Kolmogorov–Smirnov test. The color change data were analyzed by the Kruskal–Wallis test, while the iron uptake data were analyzed by one-way ANOVA followed by Tukey’s test. The level of significance was considered at *p* < 0.05.

## 3. Results

### 3.1. Results of Samples Measurement

The Kolmogorov–Smirnov test confirmed normal distribution of data regarding the amount of released iron (*p* > 0.05). One-way ANOVA was used to compare the mean amount of iron released from different iron salts, which showed a significant difference in this respect among the groups (Table 1, *p* = 0.003). Thus, pairwise comparisons were performed by Tukey’s test. As shown, the mean amount of released iron in the ferrous ammonium (*p* = 0.049) and ferrous sulfate (*p* = 0.003) groups was significantly higher than that in the control group. The mean amount of released iron in the ferrous sulfate group was also significantly higher than that in ferrous gluconate (*p* = 0.008), ferrous ammonium (*p* = 0.054), and ferrous fumarate (*p* = 0.054) groups. No other significant differences were noted (*p* > 0.05).

One-way ANOVA showed a significant difference in the mean amount of iron released from different iron salts in saline (Table 1, *p* = 0.054). Thus, pairwise comparisons were performed by Tukey’s test. As shown, the mean amount of released iron from the ferrous sulfate group was significantly higher than that in the control (*p* = 0.057), ferrous fumarate (*p* = 0.083), ferrous ammonium (*p* = 0.099), and ferrous gluconate (*p* = 0.103) groups.

An independent *t*-test was used to compare the ACC and saline groups regarding the mean amount of released iron (Table 2). The mean amount of released iron from ferrous ammonium citrate in the saline medium was significantly higher than that in the cariogenic medium. Statistical analysis indicated that there was a higher iron uptake in the ACC group when compared with saline.

### 3.2. Results of Color Change

The Kruskal–Wallis test was used to compare the color change of teeth in different groups subjected to ACC. The results showed a significant difference in color change among the five groups subjected to ACC (Table 3, *p* = 0.61). The Mann–Whitney test was applied for pairwise comparisons, which showed that the color change in all iron salt groups was significantly higher than that in the control group (*p* = 0.083 for all).

The Kruskal–Wallis test was also used to compare the color change of teeth in different saline groups. The results showed a significant difference in color change among the five saline groups (Table 3, *p* = 0.61). The Mann–Whitney test was applied for pairwise comparisons, which showed that color change in all iron salt groups was significantly higher than that in the control group (*p* = 0.083 for all).

**Figure 1:**
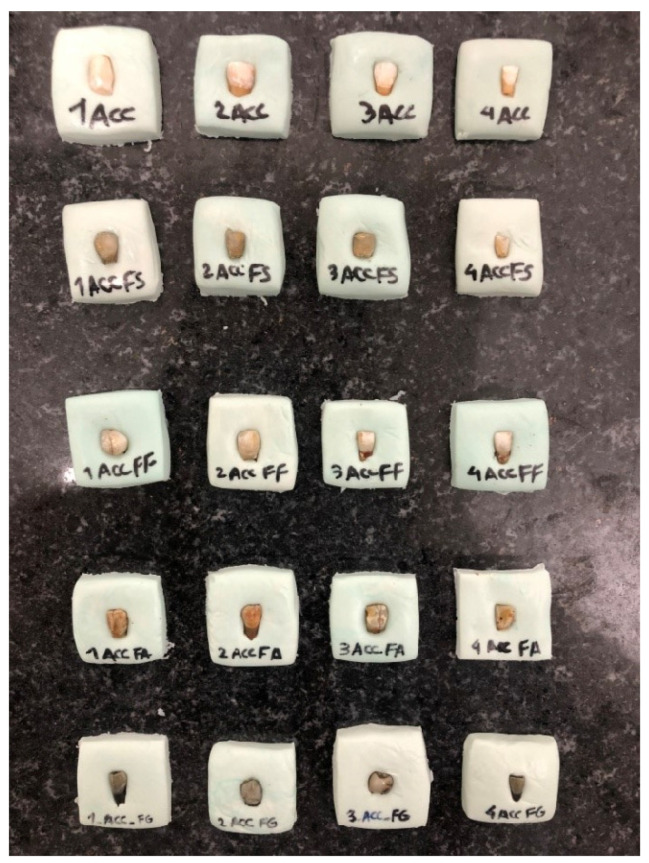
Tooth color change in ACC group.

**Figure 2:**
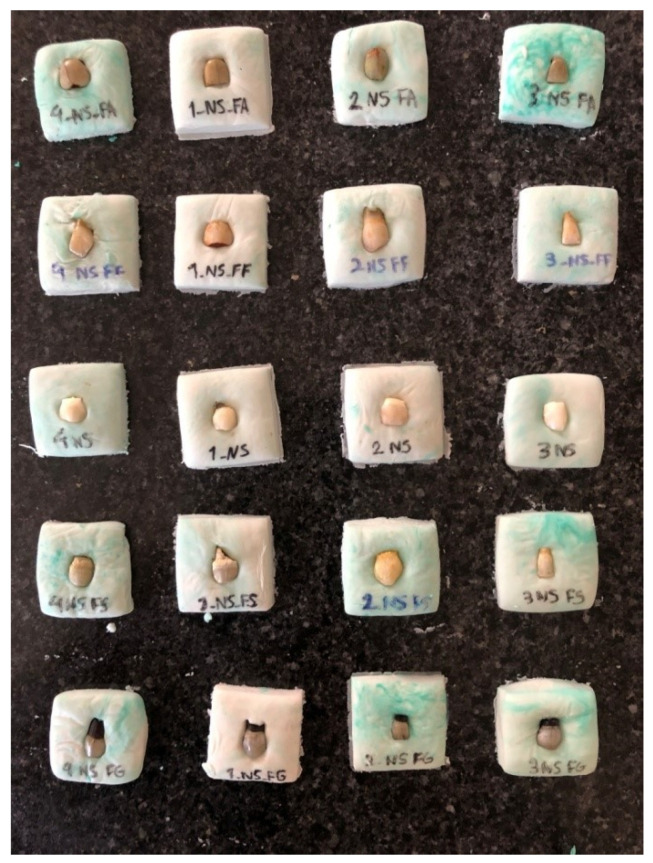
Tooth color change in normal saline group.

Pairwise comparisons of the iron salt groups subjected to ACC and immersed in saline by the Mann–Whitney test (Table 4) showed that the color change in all ACC groups was significantly higher than that in saline groups (Figure 1 and Figure 2) (*p* = 0.083 for all).

### 3.3. Structural Assessment

**Figure 3:**
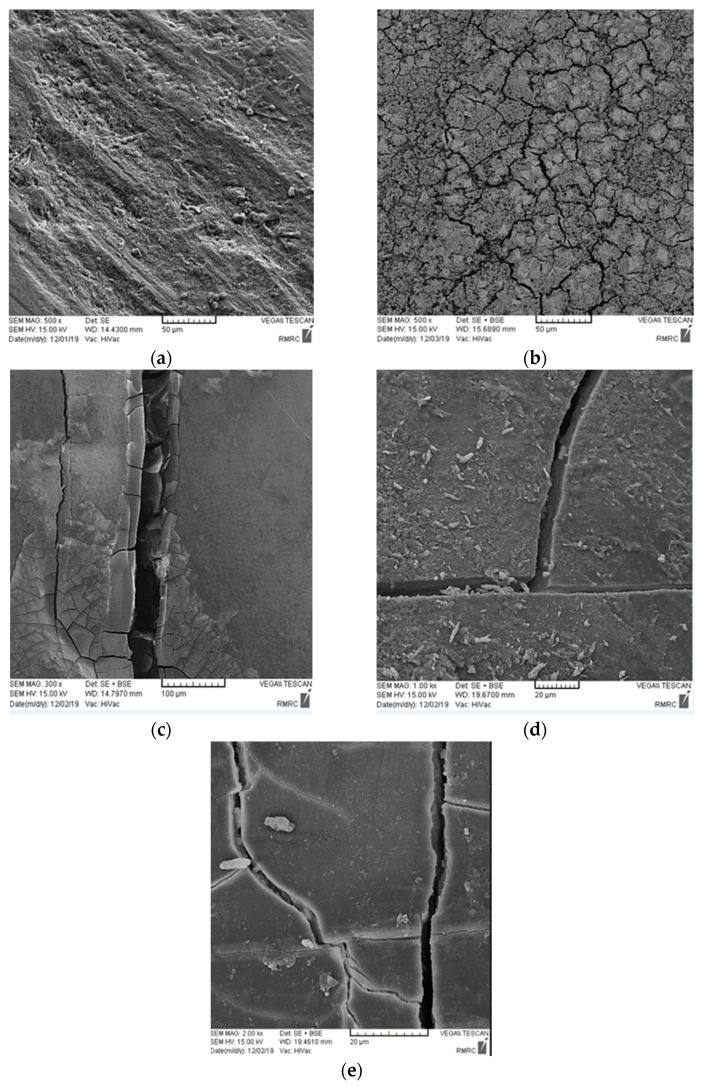
SEM micrographs of specimens exposed to different iron salts and subjected to ACC: (**a**) control group, (**b**) ferrous sulfate group, (**c**) ferrous ammonium citrate group, (**d**) ferrous fumarate group, (**e**) ferrous gluconate group.

**Figure 4:**
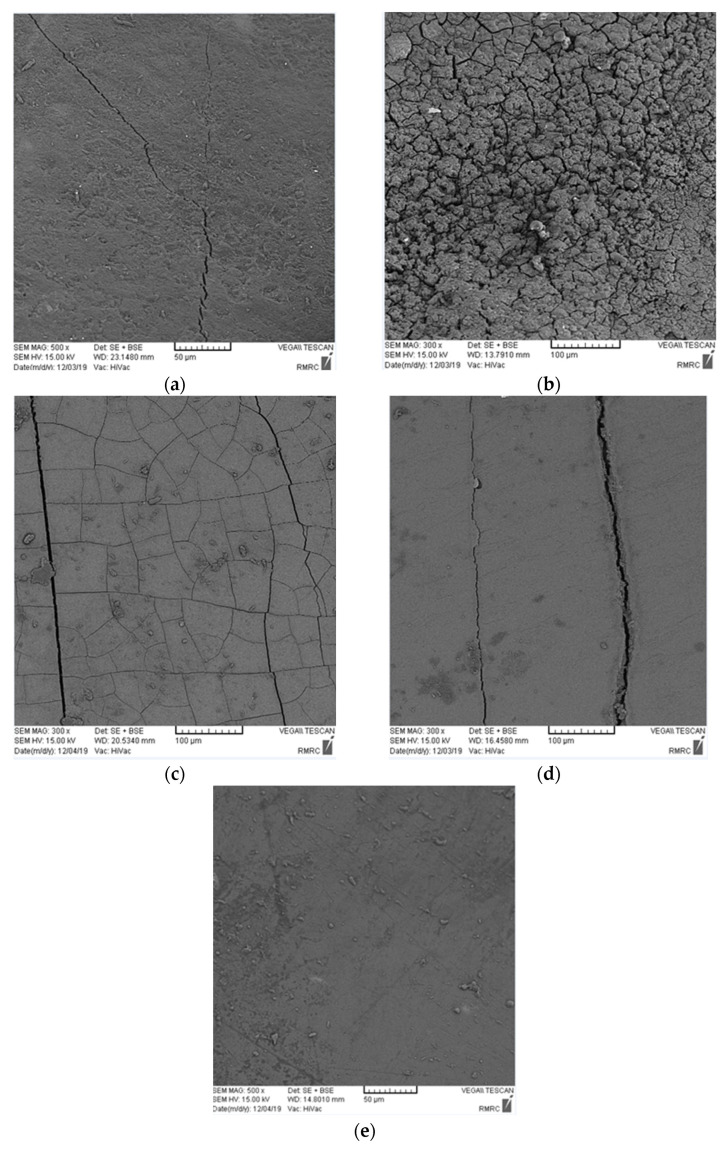
SEM micrographs of specimens exposed to different iron salts in saline: (**a**) control group, (**b**) ferrous sulfate group, (**c**) ferrous ammonium citrate group, (**d**) ferrous fumarate group, (**e**) ferrous gluconate group.

The SEM micrographs revealed that the number and depth of cracks in all four iron salt groups subjected to ACC were greater than those in the saline groups (Figure 3 and Figure 4). The number of cracks was the greatest in the ferrous sulfate group, followed by ferrous ammonium citrate, ferrous fumarate, and ferrous gluconate, in decreasing order of frequency in both media.

## 4. Discussion

This study assessed the effects of four commonly consumed iron salts on the demineralization and discoloration of primary incisor enamel subjected to ACC and saline immersion. The color change of specimens subjected to ACC was greater than that of those in saline (*p* = 0.083). The teeth subjected to ACC absorbed greater amounts of iron than did those in saline (*p* = 0.023). SEM assessment revealed a regular pattern of enamel prisms, with some broken prisms and superficial cracks in the teeth immersed in saline. The teeth subjected to ACC showed numerous fractures and cracks, which were greater in ferrous sulfate group.

Similar to the present study, Mehran et al. [1] reported the effect of iron salts on tooth color. They assessed the atomic absorption of iron, tooth discoloration, and changes in primary enamel structure caused by two different types of iron drops. They reported that teeth exposed to Kharazmi iron drops and ACC experienced greater structural changes and discoloration and had higher iron uptake. Mortazavi et al. [14] indicated black discoloration of teeth in patients who used iron supplements with high amounts of iodine, and this occurrence had a higher frequency in those with poor oral hygiene. Another study showed that optimal oral hygiene had a preventive effect on tooth discoloration following the use of iron supplements, and fluoride was also suggested for its preventive effect [15].

In the present study, the teeth subjected to ACC experienced greater discoloration than those in saline, which can be due to the impaired integrity of enamel after ACC, which would increase the contact area of the enamel with iron ions, subsequently resulting in a greater uptake of iron and higher degree of discoloration. The presence of structural defects, such as hypomineralization, decreases the enamel’s resistance to bacterial acid attacks and leads to faster and greater structural degradation, making the enamel more susceptible to discoloration [5].

Iron compounds have metal ions and thus create insoluble brown-black stains, which cause tooth discoloration. In this study, the severity of tooth discoloration was variable among different saline groups. Accordingly, ferrous sulfate caused maximum discoloration, while ferrous fumarate caused minimum discoloration. EDX analyses revealed significantly greater iron uptake by teeth subjected to ferrous sulfate, but the difference among the three other iron salt groups was not significant in terms of tooth discoloration. Tooth discoloration caused by iron is an external discoloration. Iron chemically reacts with the tooth surface and causes its black discoloration [4,5]. In addition, the greater the contact of tooth with iron, the greater the iron uptake and the resultant discoloration would be.

SEM assessments have indicated that the prevalence of caries (number and depth of cracks) is correlated with the degree of discoloration and iron uptake. Eskandarian and Joshan [15] reported that iron supplementation did not increase the frequency of caries and even decreased the rate of enamel demineralization. The same results were reported by Delbem et al. [16] and Martinhon et al. [17]. Eshghi et al. [18] and Ribeiro et al. [19] found that iron supplementation decreased the rate of caries. Thus, it may be concluded that in carious teeth, enamel porosities, loss of enamel prisms, and the large gaps formed between the enamel prisms would increase the iron uptake and subsequent discoloration. In other words, dental caries leads to greater iron uptake and greater discoloration, and iron supplementation does not lead to caries development. However, some others have reported contrary results. For instance, Tabari et al. [20] demonstrated a significant reduction in enamel microhardness as the result of exposure to iron drop. However, they did not mention the name of the iron drop, and this result may have been due to high acidity of the iron drop and subsequent enamel erosion.

The cracking of teeth can also be attributed to factors such as the crystallization behavior of iron salts, tooth dehydration when exposed to air, and replacement of calcium with iron. The four iron salts evaluated in this study have different crystallization behaviors, which may explain the differences in the iron uptake and structure of enamel in the different groups. Structural differences among the groups can also be due to the different pH values of iron drops. Ferrous sulfate is more acidic than the other three, which may explain the greater structural changes in this group. The SEM results revealed a greater depth of cracks in teeth subjected to ACC compared with those immersed in saline, which was expected, considering tooth demineralization in ACC; this finding was in line with the results of Mehran et al. [1]. Iron in very high doses protects the enamel surface from bacterial acid attacks since it forms a hydrous ferric oxide layer on the tooth surface. A previous study showed the high affinity of this layer for calcium and phosphorus in the saliva [21]. Due to an increase in the calcium and phosphorous content of this layer, the tooth surface is protected against acid attacks and erosion, and remineralization may be induced. However, such cariostatic effects depend on the amount of iron [21]. Pani et al. [22] showed that tooth discoloration due to iron exposure was greater compared with exposure to different iron compounds with an equal iron dosage. This finding may be due to the difference in the speed of iron uptake in the form of ferric and ferrous. Ferrous has a faster solubility rate than ferric; thus, it causes less discoloration. The compounds used in the present study had the ferrous form of iron. The solubility of ferrous sulfate is higher than the other three iron salts. Tooth discoloration by iron was significantly greater in ferrous sulfate group in this study. Thus, it may be concluded that the lower the solubility of the iron salt, the lower the release of iron and the lower the discoloration would be. In other words, tooth discoloration depends on the amount of iron ions and free iron in the oral environment.

Although some studies emphasized that silver diamine fluoride solution stains caries black, and the children and their parents must be well informed before application, the influence of diet on staining offers multiple opportunities for future research [23]

The manufacturers of iron drops are advised to use formulations that do not release iron into the oral environment. To achieve this goal, the affinity of iron for the elements in the complex should be higher than its affinity for calcium in the tooth structure. In addition, it should not cause any gastrointestinal problems and should be released upon exposure to stomach acid.

## 5. Limitations and Suggestions for Future Studies

We emphasize that, according to the little difference in composition observed between saliva and normal saline, it is suggested that future studies add artificial saliva to their experiments. Moreover, items such as vitamin C can be investigated as an addition, due to increased absorption of iron when accompanied with vitamin C.

## 6. Conclusions

ACC increased the structural porosities of the teeth and led to greater iron uptake and, consequently, higher discoloration. The maximum structural changes and subsequent staining occurred in the ferrous sulfate group followed by ferrous ammonium citrate, ferrous fumarate, and ferrous gluconate.

Therefore, it is mandatory to consider implementing a uniform global policy for improving the quality of the iron supplements and the benefit of their considerable effects, although there is no scientific evidence that they play a key role in dental prevention.

## Figures and Tables

**Table 1:** Mean amount of iron released from different iron salts in the experimental groups subjected to ACC and saline immersion.

Environment	Group	Mean (SD *)	Min	Max	Fisher’s Statistic	*p*-Value
ACC	Control	1.71 (1.2)	0.97	2.64	18.32	0.003
	Ferrous gluconate	6.82 (2.4)	4.23	8.91		
	Ferrous fumarate	8.79 (4.5)	4.16	14.49		
	Ferrous ammonium citrate	13.99 (2.1)	11.58	14.87		
	Ferrous sulfate	25.98 (3.7)	22.68	26.81		
Saline	Control	1.16 (0.8)	0.9	1.62	4.96	0.054
	Ferrous fumarate	2.85 (1.9)	2.10	3.21		
	Ferrous ammonium citrate	3.61 (0.7)	2.95	4.13		
	Ferrous gluconate	3.80 (0.1)	3.85	3.92		
	Ferrous sulfate	17.99 (9.46)	12.41	25.68		

* SD: Standard deviation.

**Table 2:** Comparison of the mean amount of released iron from each iron salt in the saline and cariogenic media.

Groups	NS	ACC	Independent *t*-Test Statistic	Confidence Interval		*p* Value
	Mean (SD)	Mean (SD)				
Control	1.16 (0.8)	1.71 (1.2)	1.470	−0.75	1.60	0.379
Ferrous fumarate	2.85 (1.9)	8.79 (4.5)	1.710	−0.81	1.80	0.229
Ferrous ammonium citrate	3.61 (0.7)	13.99 (2.1)	6.436	4.52	7.96	0.023
Ferrous gluconate	3.80 (0.1)	6.82 (2.4)	−1.749	−0.91	0.58	0.223
Ferrous sulfate	17.99 (9.46)	25.98 (3.7)	1.112	−0.72	0.69	0.382

**Table 3:** Color change of the teeth in different groups subjected to ACC and saline immersion.

Environment	Group	Mean Rank	Min	Max	Kruskal-Wallis Statistic	*p*-Value
ACC	Control	1.5	1	3	9.00	0.061
	Ferrous gluconate	6.5	1	8		
	Ferrous fumarate	6.5	2	8		
	Ferrous ammonium citrate	6.5	1	7		
	Ferrous sulfate	6.5	1	8		
Saline	Control	1.5	1	4	9.00	0.061
	Ferrous fumarate	6.5	2	8		
	Ferrous ammonium citrate	6.5	1	9		
	Ferrous gluconate	6.5	2	8		
	Ferrous sulfate	6.5	1	7		

**Table 4:** Comparison of the color change of the teeth in different iron salt groups subjected to ACC and saline immersion.

Group	Saline	ACC	Min	Max	Mann-Whitney Statistic	*p*-Value
	Mean Rank	Mean Rank				
Control	1.5	3.5	1	5	0.001	0.083
Ferrous fumarate	1.5	3.5	2	5	0.001	0.083
Ferrous ammonium citrate	1.5	3.5	1	6	0.001	0.083
Ferrous gluconate	1.5	3.5	1	7	0.001	0.083
Ferrous sulfate	1.5	3.5	2	6	0.001	0.083

## Data Availability

All data can be provided by the corresponding author upon reasonable request.
